# Prediction of the Prognosis of Clear Cell Renal Cell Carcinoma by Cuproptosis-Related lncRNA Signals Based on Machine Learning and Construction of ceRNA Network

**DOI:** 10.1155/2023/4643792

**Published:** 2023-03-13

**Authors:** Zhiliang Xiao, Menglei Zhang, Zhenduo Shi, Guanghui Zang, Qing Liang, Lin Hao, Yang Dong, Kun Pang, Yabin Wang, Conghui Han

**Affiliations:** ^1^School of Medicine, Jiangsu University, Zhenjiang, China; ^2^Department of Obstetrics and Gynecology, The First Affiliated Hospital of Nanchang University, Nanchang, China; ^3^Department of Urology, The Affiliated School of Clinical Medicine of Xuzhou Medical University, Xuzhou Central Hospital, Xuzhou, China

## Abstract

**Background:**

Clear cell renal cell carcinoma's (ccRCC) occurrence and development are strongly linked to the metabolic reprogramming of tumors, and thus far, neither its prognosis nor treatment has achieved satisfying clinical outcomes.

**Methods:**

The Cancer Genome Atlas (TCGA) and Gene Expression Omnibus (GEO) databases, respectively, provided us with information on the RNA expression of ccRCC patients and their clinical data. Cuproptosis-related genes (CRGS) were discovered in recent massive research. With the help of log-rank testing and univariate Cox analysis, the prognostic significance of CRGS was examined. Different cuproptosis subtypes were identified using consensus clustering analysis, and GSVA was used to further investigate the likely signaling pathways between various subtypes. Univariate Cox, least absolute shrinkage and selection operator (Lasso), random forest (RF), and multivariate stepwise Cox regression analysis were used to build prognostic models. After that, the models were verified by means of the C index, Kaplan–Meier (K-M) survival curves, and time-dependent receiver operating characteristic (ROC) curves. The association between prognostic models and the tumor immune microenvironment as well as the relationship between prognostic models and immunotherapy were next examined using ssGSEA and TIDE analysis. Four online prediction websites-Mircode, MiRDB, MiRTarBase, and TargetScan-were used to build a lncRNA-miRNA-mRNA ceRNA network.

**Results:**

By consensus clustering, two subgroups of cuproptosis were identified that represented distinct prognostic and immunological microenvironments.

**Conclusion:**

A prognostic risk model with 13 CR-lncRNAs was developed. The immune microenvironment and responsiveness to immunotherapy are substantially connected with the model, which may reliably predict the prognosis of patients with ccRCC.

## 1. Introduction

CCRCC is the most prevalent subtype of renal malignancy, accounting for nearly 70% of all cases [1]. In addition, it exhibits higher rates of recurrence, metastasis, and mortality when compared to chromophobe cell renal carcinoma (cRCC) and papillary renal cell carcinoma (pRCC) [2, 3]. Due to the insidious nature of ccRCC, 30% of patients have metastases when they are first diagnosed [4]. Currently, partial or radical nephrectomy is the best treatment option for nonmetastatic ccRCC patients, but this procedure has a postoperative recurrence rate that can range from 20 to 40%, which has a substantial impact on patient prognosis [5]. In addition, radiation and chemotherapy frequently have poor results for patients with metastatic ccRCC, and drug resistance brought on by prolonged medication frequently results in a terrible prognosis. Despite the fact that immunotherapies such as programmed death-1 (PD-1) and programmed death ligand 1 (PD-L1) have been employed in the treatment of ccRCC recently and have demonstrated some therapeutic results, some patients still do not respond well to this course of action [6, 7].

According to previous research, copper can induce tumor angiogenesis, which aids in the progression of cancer, as well as be directly linked to the occurrence and growth of a variety of malignancies [8–10]. Besides that, certain outcomes have been attained previously based on the use of copper ion chelators in the therapy of cancer [11, 12]. The key to pathological and physiological processes is cell death, and cuproptosis is the most recent type of death that differs from previous cell deaths such as apoptosis [13], necrosis [14], and ferroptosis [15]. According to the research, iron-sulfur cluster protein loss and fatty acylated protein aggregation are induced by copper binding to tricarboxylic acid (TCA) cycle fatty acyl proteins, which results in death from toxic protein stress [16]. In this regard, it is worth noting that studies have shown that the occurrence and development of ccRCC frequently involve reprogramming of the TCA cycle. This is primarily accomplished by affecting the upregulation of the VHL/HIF pathway, which results in the inhibition of the TCA cycle, thereby promoting the occurrence and development of ccRCC [17–19]. In view of this, the cuproptosis theory may provide a novel approach to the therapy of ccRCC.

Long noncoding RNA (LncRNA) is a subclass of noncoding RNAs that can take part in and control a number of pathophysiological processes. lncRNA is a noncoding RNA whose biological function is more than 209 bases long. Similar to coding genes, lncRNAs can be chromatin reprogrammed. Dysregulation and posttranscriptional regulation of enhancers are widely involved in biological, physiological, and pathological processes. As a newly discovered class of RNA molecules, several lncRNAs have been identified as biomarkers of cancer, which control tumor proliferation, immune evasion, cell death resistance, and regional or distant metastasis. Therefore, lncRNA represents an important improvement in our understanding of copper worm disease and evidence that lncRNA is a therapeutic target that can induce GC copper Fibrobacteres. However, the specific role of lncRNA in the adjustment of aeruginosa is largely unknown. By controlling metabolic reprogramming, lncRNA can regulate carcinogenesis [20]. Additionally, studies have shown that lncRNAs play a variety of roles in the development of ccRCC, including upregulating lncRNA PVT1 and activating the HIF2*α* pathway to promote the growth and progression of ccRCC cells, as well as lncRNA HCG18, which promotes ccRCC migration and transfer by modulating the miR-152-3/RAB14 axis [21, 22]. LncRNA can also be used to predict the progression of ccRCC [23, 24]. In a recent study, it was found that CRGS is linked to immune infiltration and the immune checkpoint PD-1, which can help predict how well ccRCC patients would fare and offer new information about how to treat the disease [25]. Nevertheless, there is still a lack of knowledge about the mechanism of action of CR-lncRNA in ccRCC, particularly its influence on prognosis. This study investigated the function of CR-lncRNA in ccRCC and developed a new prognostic model based on CR-lncRNA, which may offer fresh perspectives for future studies on ccRCC and patient-specific management.

The proposed CR-lncRNA-based prognostic model includes the following advantages. (1) Due to the discrete Fourier transform data, its main information components are concentrated in the low-frequency part of the frequency domain, and the high-frequency part is mainly secondary information or noise. Therefore, the lengthening lncRNA sequence can be truncated into a fixed-length vector by intercepting the fixed-length part of the low frequency. (2) Two traditional convolutional models were used vgg16_bn build task models with Resnet18. Firstly, to adapt the data dimension, the commonly used two-dimensional convolution and pooling are adjusted to one-dimensional convolution and pooling. At the same time, since the label data are a twenty-four-dimensional data, the task model is extended to a multioutput model. LncRNA tissue-specific analysis was performed on multiple output regression, multiple output classification, and multilabel classification, respectively.

## 2. Methods and Materials

### 2.1. Data Collection

A recent significant study investigated the cuproptosis subtypes and built a predictive model to improve the prognosis of patients with CRC. Gene expression data were downloaded from the TCGA database to identify distinct molecular subtypes using a nonnegative matrix factorization algorithm [16]. Samples with a survival time of less than 30 days were disregarded as we downloaded the gene expression profile data, clinical information data, mutation data, and copy number variation (CNV) data of ccRCC from the TCGA official website. Finally, 71 normal samples and 511 tumor samples were comprised. The GEO database provided the CRGS gene expression profile in ccRCC. Gene count values were employed for differential analysis, and for downstream analysis, count values were converted to log2 (TPM +1) values.

### 2.2. Analysis of Genetic Mutation Data of CRGS

The TCGA Kidney Renal Clear Cell Carcinoma (TCGA-KIRC) cohort was used to investigate the differences in CRGS expression between normal and malignant samples. These discrepancies in gene expression were then re-examined in the GSE53757 and GSE40435 cohorts. We further confirmed them using the immunohistochemistry results of proteins in the Human Protein Atlas (HPA) database to assess their alterations in protein expression. The location of these genes on different chromosomes was visualized using the “RCircos” package, the “maftools” package was used to plot the mutational landscape of these genes, and finally, univariate COX regression analysis and log-rank test were performed to investigate the impact of these genes on the prognosis of ccRCC patients.

### 2.3. Consensus Clustering Analysis Based on CRGS

Using the “ConsensusClusterPlus” R package, we performed unsupervised clustering of the ccRCC samples based on the expression patterns of the 19 CRGS. To ensure the stability of the clusters, 1000 random repeated samplings were carried out on 80% of the samples and all genes. The Euclidean distance clustering algorithm was selected. The appropriate number of clusters was established through using cumulative distribution function (CDF) and intra-group correlation. To confirm the discriminating of various subtypes, principal component analysis (PCA) was utilized. The variations in survival among various subtypes were then shown using K-M survival curves, and the log-rank test was used to determine whether the differences were statistically significant.

### 2.4. Identification of Molecular Characteristics, Immune Infiltration Characteristics, and Immunotherapeutic Response Based on Different Subtypes

The “GSVA” package was used to study the pathways implicated in various subtypes through gene set variation analysis (GSVA). To further investigate the differences in immune infiltration features across distinct subtypes, the infiltration abundance of diverse immune cells in different subtypes was estimated using the single sample genes enrichment analysis (ssGSEA) algorithm of the aforementioned R package. The tumor immune dysfunction and exclusion (TIDE) approach, which was developed in recent years, can be used to anticipate whether immunotherapy will benefit tumor patients. This research thorough investigation of hundreds of distinct tumor expression profiles looked for indicators to predict whether patients would respond to immune checkpoint blockade (ICB) therapy, i.e., a higher TIDE score indicates a lower likelihood of responding to immunotherapy [26]. The website it created (https://tide.dfci.harvard.edu/) was subsequently used to forecast the immunotherapy response in patients from different subtypes. The results of the analysis were visualized using the R packages “tinyarray,” “pheatmap,” and “ggplot2.” Statistical significance was set at a *P*-value <0.05.

### 2.5. Differential Analysis of mRNA, lncRNA, and MicroRNA

The three approaches of “Edger,” “DESeq2,” and “limma” were utilized to produce the overlapping mRNAs, lncRNAs, and microRNAs (miRNAs), which were then employed as the differential genes. |Log2fold change| >1 and false discovery rate (FDR) <0.05 were the screening criteria thresholds for the approaches described above.

### 2.6. WGCNA Identifies Cuproptosis-Related Modules

The “WGCNA” R package was used to conduct the WGCNA analysis of the lncRNAs from the ccRCC samples. According to the scale-free network criteria, the best soft threshold was chosen. The modules with distances of less than 0.25 were then combined, and the minimum number of genes for the modules was set at 30. The modules having the strongest association with cuproptosis were chosen for further analysis after a correlation analysis between the modules and cuproptosis phenotypic data was completed.

### 2.7. Construction and Evaluation of Prognostic Risk Scoring Models

The R package “Caret” was used to first randomly divide the ccRCC samples in the TCGA queue into training and test sets in a ratio of 7 : 3. The training set and test set are used to train and test the model, respectively, to create a stable model. After intersecting the differential lncRNA of the ccRCC with the lncRNA in the module most associated with cuproptosis discovered by the aforementioned WGCNA, a univariate Cox regression analysis and log-rank test were carried out. Subsequently, lncRNAs with a *P* value <0.05 obtained by both of the above two test methods were considered as candidate lncRNAs. We selected characteristic genes using two machine learning approaches, namely, Lasso regression and RF, to avoid the overfitting of the model. A well-known machine learning technique called lasso regression decreases the dimensionality of high-dimensional data by assigning each feature a penalty coefficient that makes the coefficient of unimportant features 0 and therefore eliminates collinearity across features. Is frequently employed to tune the COX proportional hazards model (CPH) [27]. Studies have proven that RF can also be used to model survival analyses, and a minimal depth (MD) strategy has also been developed to identify key prognostic characteristics, and recent studies have pointed out that tree-based machine learning methods outperform deep learning in dealing with tabular data [28–30]. The overlapping lncRNAs chosen by the two machine learning methods above were subjected to multivariate stepwise Cox regression analysis, and the best CPH model was found according to the Akaike information criterion (AIC) criteria, which states that the smaller the AIC value, the better the model's performance [31]. The performance and precision of the model in the training set were assessed by using the time-dependent ROC curve and the C-index, and the performance of the risk score as an independent prognostic indicator was confirmed utilizing univariate and multivariate Cox regression analysis. In order to more thoroughly assess the performance of our prognostic model, we gathered a number of lncRNA-based prognostic risk scoring models developed based on the TCGA-KIRC cohort in recent years, computed the time-dependent ROC curve and C index of the lncRNA model based on the entire TCGA-KIRC cohort, and compared them with our developed prognostic model [32–37].

### 2.8. Construction of Competing Endogenous RNA (ceRNA) Network

We predicted the target miRNAs of these lncRNAs by applying the mircode website (https://mircode.org/download.php) based on the overlapping lncRNAs identified by the aforementioned univariate Cox analysis and log-rank test. The resulting target miRNAs were crossed with the differential miRNAs and then submitted to miRDB (https://mirdb.org/), miRTarBase (https://mirtarbase.cuhk.edu.cn/), and targets can (https://www.targetscan.org/). The target mRNAs of the aforementioned miRNAs were predicted by the three websites in turn. The final target mRNAs were chosen based on predictions made simultaneously by the three websites' predicted targets. We created a lncRNA-miRNA-mRNA ceRNA network in the Cytoscape based on the above predicted results.

### 2.9. Functional Enrichment Analysis

Using the LNCSEA online platform (https://bio.liclab.net/LncSEA/), the functional enrichment analysis of the aforementioned prognostic CR-lncRNAs was carried out [38]. Functional enrichment annotation of CR-lncRNA target miRNAs was performed using the miEAA online tool (https://ccb-compute2.cs.uni-saarland.de/mieaa2/) [39]. In order to investigate the probable biological pathways of patients in different risk groups, we simultaneously performed gene set enrichment analysis (GSEA) on patients in high- and low-risk groups using the R package “clusterProfiler.” The adjusted *P*value <0.05 and *q* value <0.05 were used to identify statistically significant enriched pathways.

### 2.10. Immunotherapy Response Prediction and Drug Sensitivity Analysis

ICB therapy has now been proven to be beneficial for some tumor patients, although the majority of patients do not gain from it, which may be partially attributed to tumor heterogeneity and varied immune checkpoint expression. In order to determine whether patients might benefit from immunotherapy, we compared the immune checkpoint expression between high- and low-risk patients. We then used the TIDE website to estimate the immunotherapy response for ccRCC patients in different risk groups. Sensitive anticancer drugs were examined using the “pRRophetic” R package for two risk groups. The Wilcoxon rank test was used to analyze differences between various risk groups, and a *P* value lower than 0.05 was regarded as statistically significant.

### 2.11. Statistical Analysis

R software was used to perform all analyses (version 4.1.2). The association analysis of two categorical variables and the sample rate (composition ratio) of two or more groups were both compared using the chi-square test. To determine whether there were any differences in the distribution of measurement data or grade data between the two groups, a Wilcoxon rank-sum test was utilized. The Kruskal–Wallis test was used for nonparametric comparisons when there were three or more groups. For correlation testing in the correlation analysis, Spearman, and distance correlation tests were employed. The statistical significance was defined as a *P* value of 0.05, where ^*∗*^ denotes *P* value <0.05, ^*∗∗*^ denotes *P* value <0.01 and ^*∗∗∗*^ denotes *P* value <0.001, and ns denotes no statistical significance.

## 3. Results

### 3.1. Landscape of Genetic Mutations in CRGS

**Figure 1:**
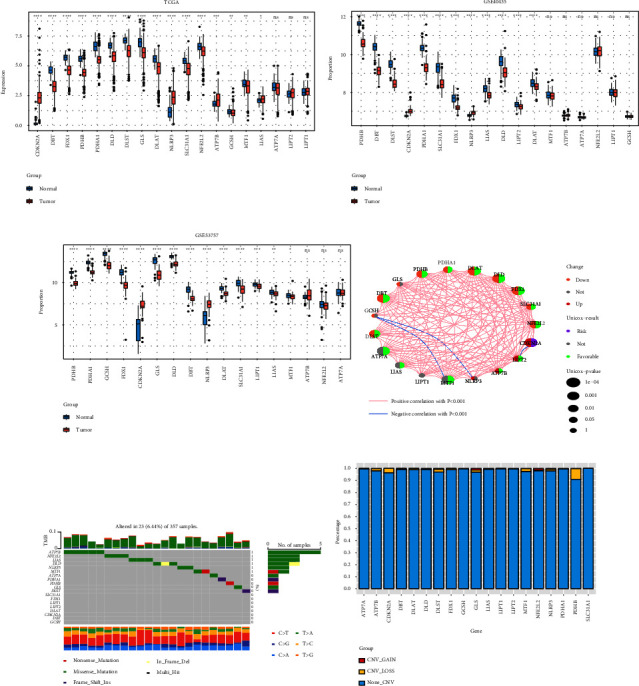
Identification of 19 CRGS and their genetic mutational landscape. (a) Differential expression of CRGS in the TCGA cohort; (b) differential expression of CRGS in the GSE40435 cohort; (c) differential expression of CRGS in the GSE53757 cohort; (d) co-expression network between CRGS; (e) mutation frequency of CRGS; (f) copy number variation frequency of CRGS. ^*∗*^*P* value <0.05, ^*∗∗*^*P* value <0.01, ^*∗∗∗*^*P* value <0.001, ^*∗∗∗∗*^*P* value <0.0001.

The 19 CRGS were acquired through recent significant scientific discoveries [16]. Following that, differential analysis of the previously mentioned genes in the TCGA cohort revealed that, with the exception of LIPT1, LIPT2, and ATP7A, which revealed no statistically significant differences, the expression of the majority of CRGS differed significantly between normal and tumor samples and most of them were downregulated (Figure 1(a)). Next, the expression levels of these CRGS were checked again in the two GEO cohorts, GSE40435 and GSE53757, and although the results were slightly different from the TCGA cohort, the general results were similar (Figures 1(b) and 1(c)). It is common knowledge that proteins carry out the majority of biological processes in humans. To this end, we further assessed the variance in these genes protein expression in the HPA database between tumor and normal tissues. The outcomes demonstrated that most genes expressed differently at the protein level as well (Figure 1(f)). The findings from the K-M survival curve were similar to those from the univariate Cox analysis, with the exception that CDKN2A and GCSH were risk factors, whereas the remaining genes were protective (Figure 1(d)). The somatic mutation rate of each CRGS was incredibly low, and just 23 (6.44%) of 357 ccRCC samples showed genetic alterations, according to our analysis of somatic mutations in these genes (Figure 1(e)). Figure 1(f) demonstrates that the majority of CRGS have low CNV frequencies. The frequency of copy number deletions is almost 9% for only PDHB. On the chromosome, CRGS is located, as shown in Figure 1(c). We hypothesized that the genetic variation in ccRCC is largely stable because both somatic mutations and CNV frequencies had very small sample sizes. Of course, additional elements such as methylation and histone modifications might also be at work. According to the aforementioned findings, CRGS has a significant impact on the prognosis of ccRCC patients as well as the occurrence and progression of cancer.

### 3.2. Identification of Molecular Subtypes Based on CRGS

**Figure 2:**
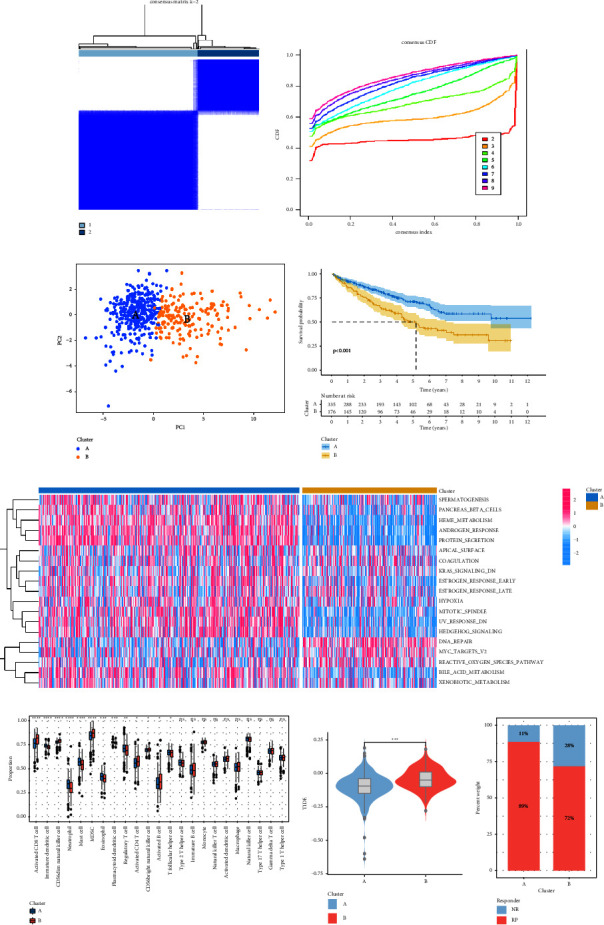
Consensus cluster analysis, immune infiltration analysis, and immunotherapy response analysis based on 19 CRGS. (a) Consensus clustering-based on CRGS; (b) cumulative distribution function plot; (c) PCA analysis between two cuproptosis clusters; (d) survival analysis between two cuproptosis clusters; (e) GSVA analysis; (f) differences between 23 immune cells in different cuproptosis clusters; (g) differences in TIDE scores among different cuproptosis clusters; and (h) differences in immunotherapy response among different cuproptosis clusters. ^*∗*^*P* value <0.05, ^*∗∗*^*P* value <0.01, ^*∗∗∗*^*P* value <0.001, ^*∗∗∗∗*^*P* value <0.0001.

We used the “ConsensusClusterPlus” R package, a consensus clustering method based on a machine learning algorithm, to perform unsupervised clustering of ccRCC patients-based on the expression levels of the 19 CRGS. Finally, as shown in Figures 2(a) and 2(b), we were able to distinguish the cuproptosis molecules into two optimum clusters, A and B, each of which had 335 and 176 samples, respectively. Based on the abovementioned results, we can infer that patients in clusters A and B reflect two distinct cuproptosis phenotypes, with cluster A presenting the activating subtype of cuproptosis and cluster B representing the suppressing subtype. The PCA results demonstrated good discrimination between the two distinct subtypes (Figure 2(c)). A subsequent study of survival analysis revealed that patients in cluster A had significantly higher overall survival (OS) than those in cluster B (Figure 2(d)). For the two subtypes, GSVA analysis identified separate underlying biological processes and pathways (Figure 2(e)). The pathways DNA repair, Myc targets, Reactive Oxygen Species pathway, and Kras Signaling pathway, which are typically linked to tumor development and tumor immune inflammation, were significantly enriched in patients in cluster B compared with patients in cluster A. Therefore, the reason that cluster B patients have a poor prognosis may be due to the activation of the aforementioned pathways. However, the spermatogenesis, pancreas beta cells, heme metabolism, and androgen response of patients in cluster A were significantly enriched. In light of the variations in the biological pathways mentioned above, we investigated the immune infiltration traits of the two subtypes. As can be seen in Figure 2(f), cluster A had a larger concentration of infiltrating neutrophil, mast, and eosinophil cells, whereas cluster B had a higher concentration of infiltrating activated CD8 T cells, CD4 T cells, activated B cells, and myeloid-derived suppressor cells (MDSC) cells. Then, using the TIDE website, we predicted whether certain patient subgroups would respond to immunotherapy. According to Figure 2(g), patients in cluster A had lower TIDE scores, making them more likely to benefit from immunotherapy.  Figure 2(h) compares the immunotherapy responses of different patient subgroups (cluster A, 89% vs. cluster B, 72%). Our findings imply that therapeutic regimens developed for cuproptosis may be a potential anticancer target in ccRCC patients and may improve ccRCC patients' responsiveness to immunotherapy.

### 3.3. Identification of CR-lncRNAs

**Figure 3:**
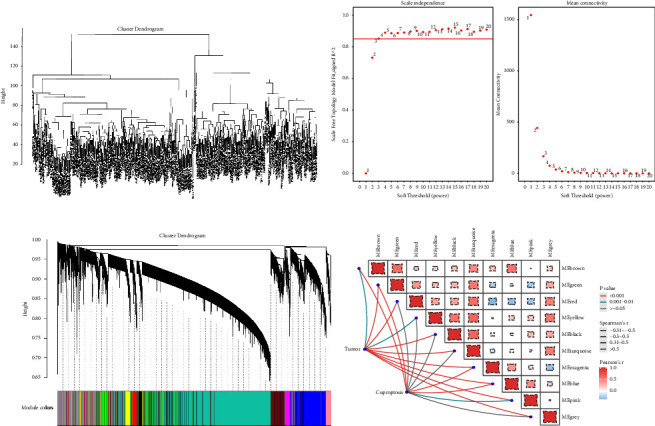
Weighted correlation network analysis. (a) Cluster analysis of all samples in the TCGA cohort; (b) analysis of network topology for various soft thresholding powers. The left panel shows the scale-free fit index (*y*-axis) as a function of the soft thresholding power (*x*-axis). The right panel displays the mean connectivity (degree, *y*-axis) as a function of the soft-thresholding power (*x*-axis); (c) clustering dendrogram of different similarity genes-based on topological overlap; and (d) the module-traits associations diagram. Each grid corresponds to a module, the color of the grid represents the size of the correlation between different modules, the thickness of the lines represents the size of the correlation between modules and phenotypes, and the color of the lines represents the size of the *P* value of the correlation test between modules and phenotypes.

As master regulators of gene expression, lncRNAs have been implicated in a number of malignancies in recent years. A notable illustration is PCAT-1 dysregulation, which is strongly linked to the development of prostate cancer [40]. Additionally, lncRNAs can be employed independently to forecast tumor prognosis, tumor progression, and disease diagnosis [41, 42]. Therefore, we retrieved the lncRNA expression profiles of ccRCC patients from the TCGA database and, after deleting the lncRNAs that were barely expressed, acquired 9024 lncRNAs for WGCNA analysis. The WGCNA network was built using the one-step method, and Figure 3(a) shows that there were no outlier samples discovered and that the samples were well clustered. The ideal soft threshold of 3 was identified using the scale-free topology fitting index of 0.85 and network connectivity as the standard (Figure 3(b)). A hierarchical clustering dendrogram that obtained 10 modules is shown in Figure 3(c). As can be seen from Figure 3(d), the blue, green, and magenta modules are all significantly associated with tumor and cuproptosis, but the blue module has the strongest correlation with tumor (*r* = −0.5, *P* < 0.001). As a result, we chose the lncRNAs identified in the blue modules to further develop the prognostic molecular characteristics of ccRCC patients.

### 3.4. Construction and Validation of Prognostic Risk Scoring Model

**Figure 4:**
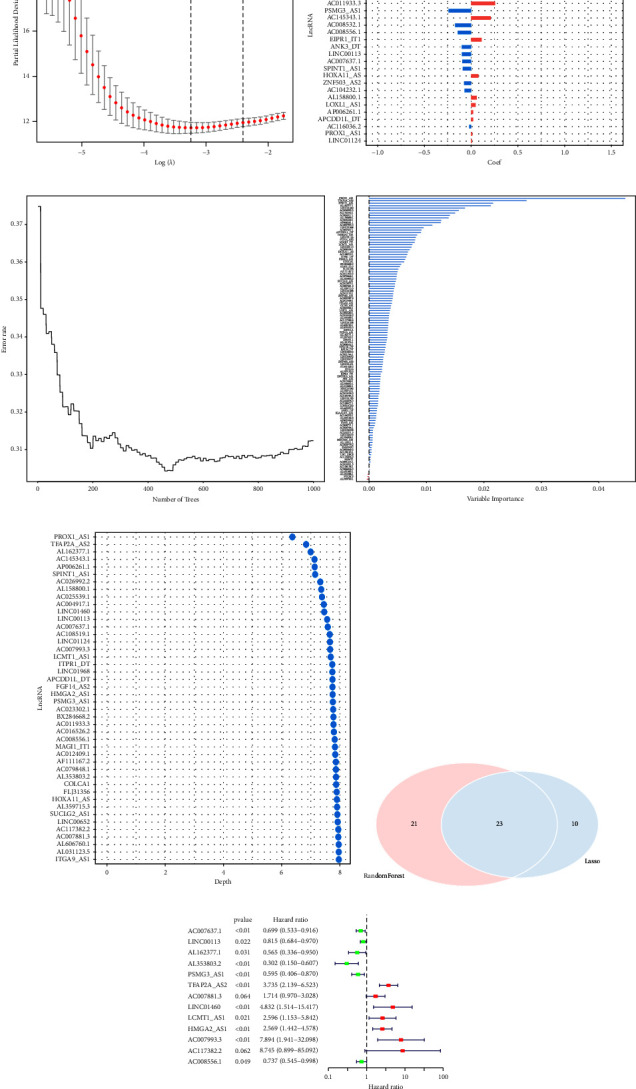
Construction of a prognostic risk scoring model. (a) Tuning parameter map for lasso regression based on 10-fold cross-validation; (b) the selected lncRNAs and their regression coefficients based on lambda with the smallest mean error of lasso regression; (c) prognostic feature lncRNAs selected based on the random forest algorithm; (d) prognostic signature lncRNAs selected based on the MD method; (e) venn diagram showing overlapping lncRNAs obtained by the lasso algorithm and random forest algorithm; (f) forest plot showing 13 CR-lncRNAs obtained by multivariate stepwise COX regression analysis.

We eventually discovered 4229 overlapping mRNAs, 2287 overlapping lncRNAs, and 181 overlapping miRNAs using the three methods of “EdgeR,” “DESeq2,” and “limma” for gene differential analysis. The differential lncRNAs were intersected with the 1033 lncRNAs in the blue module above to provide 630 overlapping lncRNAs as candidate lncRNAs. Subsequently, univariate Cox regression analysis and the log-rank test yielded 116 lncRNAs with prognostic significance (*P* value <0.05).  Figure 4(a) displays the optimum parameter (*λ*) interval for Lasso regression using 10-fold cross-validation. When we selected the *λ* value with the smallest mean error, we got 33 lncRNAs (Figure 4(b)). The relationship between the number of trees and the error rate in the RF algorithm is illustrated in Figure 4(c), along with the characteristic genes the algorithm identified. It is clear that as the tree expands, the error rate curve gradually flattens out, showing that the number of trees chosen was sound. The MD approach yielded a threshold of 7.9681, and using this threshold, we were able to derive 44 significant eigengenes (Figure 4(d)). By intersecting the lncRNAs produced by the previous two approaches, we identified 23 potential lncRNAs (Figure 4(e)). Based on the aforementioned potential lncRNAs, a multivariate stepwise CPH model was created, and with an AIC = 1234.71, we were able to generate the ideal CPH model for 13 lncRNA combinations in the training set (Figure 4(f)).

**Figure 5:**
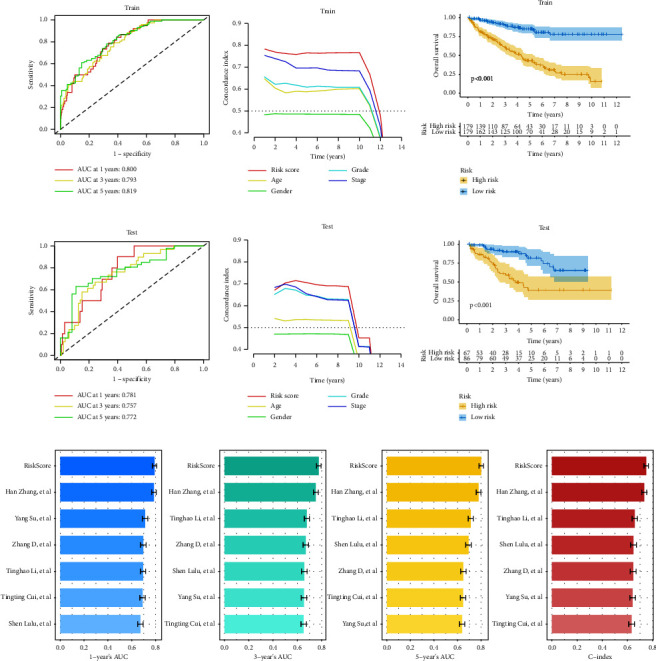
Evaluation and validation of prognostic risk scoring models. (a–c) Time-dependent ROC curves, C index, and K-M survival analysis in the training set; (d–f) time-dependent ROC curves, C index, and K-M survival analysis in the test set; (g) comparison of AUC values and C-index of different prognostic risk score models.

The expression of lncRNA in the aforementioned model and the regression coefficient obtained by multivariate stepwise Cox regression analysis were used to generate the risk score for each patient. The following is the calculating formula: risk score = (−0.3586 *∗* AC007637.1 exp) + (−0.2050 *∗* LINC00113 exp) + (−0.5718 *∗* AL162377.1 exp) + (−1.1979 *∗* AL353803.2 exp) + (−0.5197 *∗* PSMG3-AS1 exp) + (1.3177 *∗* TFAP2A-AS2 exp) + (0.5387 *∗* AC007881.3 exp) + (1.5752 *∗* LINC01460 exp) + (0.9538 *∗* LCMT1-AS1 exp) + (0.9434 *∗* HMGA2-AS1 exp) + (2.0661 *∗* AC007993.3 exp) + (2.1685 *∗* AC117382.2 exp) + (−0.3046 *∗* AC008556.1 exp). Based on the median risk score, patients were separated into high- and low-scoring groups. The risk score's area under the curve (AUC) at one year, three years, and five years is 0.800, 0.793, and 0.819, respectively, according to the ROC curve of the training set (Figure 5(a)). The ROC curve of the test set also displays greater accuracy, with AUCs exceeding 0.75 at one year, three years, and five years (Figure 5(d)). The C index, which was 0.77 in the training set (Figure 5(b)) and 0.71 in the validation set (Figure 5(e)), both of which were considerably higher than the remaining clinicopathological variables, also showed that the model had great consistency. In the training and test sets, the OS of patients in the high-score grouping was considerably lower than that in the low-risk category (Figures 5(c) and 5(f), *P* value <0.001). Additionally, the prognostic risk model we created performs better than some current models when comparing the two indicators of AUC and the C index (Figure 5(g)). The results above show that the prognostic risk score model based on 13 CR-lncRNAs can precisely predict the prognosis of ccRCC patients.

### 3.5. Correlation of Prognostic Risk Scoring Models with Clinical Pathological Features

**Figure 6:**
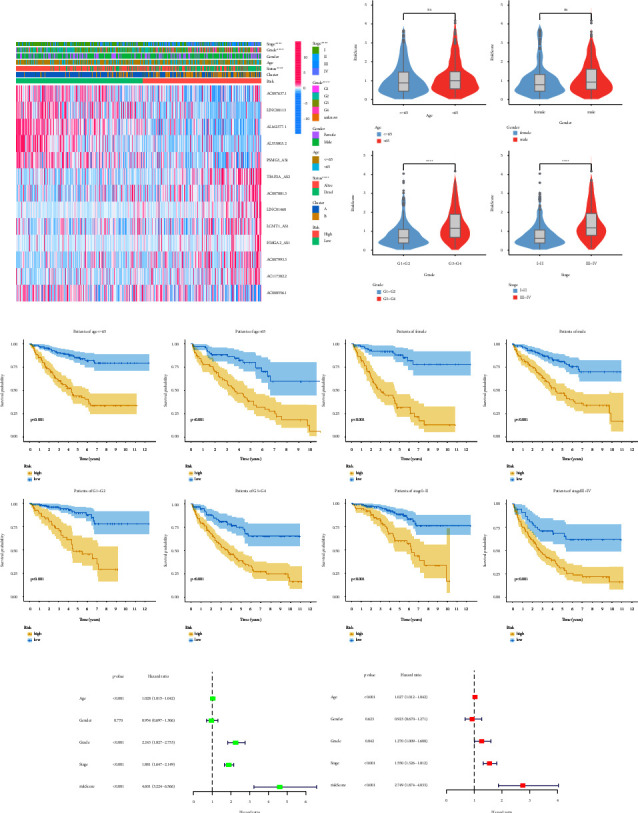
Association of prognostic risk score models with clinicopathological variables. (a) Heatmap of clinical correlations for prognostic models; (b) differences in risk scores between different clinical subgroups; (c) differences in overall survival among patients in different risk groups in different clinical subgroups; (d) univariate COX regression analysis of risk scores and clinicopathological variables; (e) multivariate COX regression analysis of risk scores and clinicopathological variables. ^*∗*^*P* value <0.05, ^*∗∗*^*P* value <0.01, ^*∗∗∗*^*P* value <0.001, ^*∗∗∗∗*^*P* value <0.0001.

The association between risk scores and clinicopathological characteristics was also demonstrated by our investigation. As observed in Figure 6(a), grade and stage vary among various risk groups even if risk scores are really not related to age and gender. Furthermore, a greater risk score was significantly correlated with both a higher grade and stage (Figure 6(b)). Likewise, the outcomes of patients in the high-risk group were considerably worse than those in the low-risk group in all clinical subgroups, according to the findings of the subsequent survival analysis (Figure 6(c)). The risk score was also revealed to be an independent prognostic factor in ccRCC patients by univariate and multivariate Cox regression analysis (Figures 6(d) and 6(e)). In light of the aforementioned findings, the prognostic risk score model, which is made up of 13 CR-lncRNAs, is a very promising biomarker that can not only accurately predict the prognosis of ccRCC patients but also assess their clinical progression.

### 3.6. Correlation of Prognostic Models with Tumor Immune Microenvironment and Immunotherapy Responses

**Figure 7:**
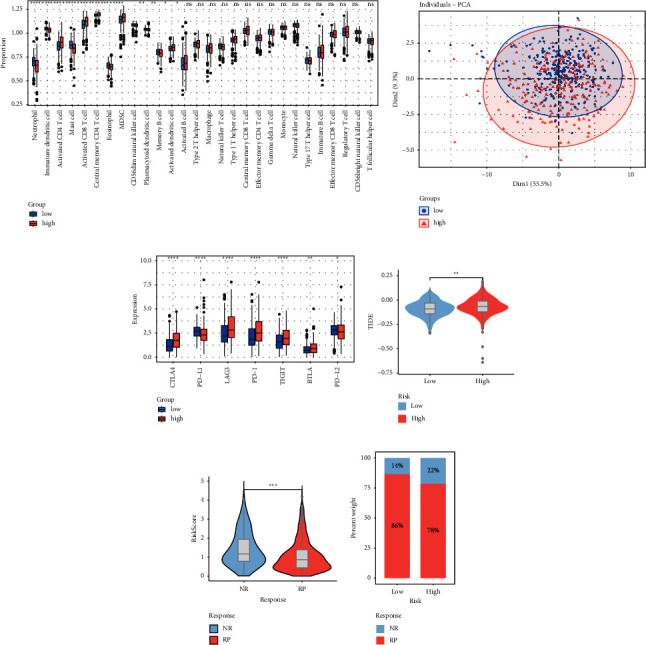
Correlation of prognostic risk score models with immune infiltration and response to immunotherapy. (a) Differences in immune cell infiltration between patients in high and low-risk groups; (b) PCA analysis reveals distinct immune microenvironments between different risk groups; (c) differences in the expression of immune checkpoints between high and low-risk groups; (d) differences in TIDE scores between high and low-risk groups; (e-f) differences in immunotherapy response between high and low-risk groups. ^*∗*^*P* value <0.05, ^*∗∗*^*P* value <0.01, ^*∗∗∗*^*P* value <0.001, ^*∗∗∗∗*^*P* value <0.0001.

The ssGSEA analysis suggests that the immune infiltration features of the patients in the two risk groups varied. While patients in the low-risk group had higher rates of neutrophil, immature dendritic cell, and mast cell infiltration, patients in the high-risk group had higher rates of activated CD4 T cell, activated CD8 T cell, and MDSC infiltration (Figure 7(a)). It was further revealed by PCA analysis that the two patient groups represented various immune cell infiltration microenvironments (Figure 7(b)). The majority of immunological checkpoints were more strongly expressed in the high-risk group, whereas PD-L1 and PD-L2 expression were more prominent in the low-risk group (Figure 7(c)). According to the current understanding, immunotherapy has a greater chance of helping tumor patients the more PD-L1 is expressed. Additionally, because the majority of immune checkpoints are significantly expressed in the high-risk group, it is more likely to produce immunosuppression, which will cause the cancer to advance in those people. We next used the TIDE online tool to predict immunotherapy responses for patients in the two groups once more. The findings demonstrated that the low-risk group had lower TIDE scores than the high-risk group (Figure 7(d)). Moreover, Figures 7(e) and 7(f) show that better immunotherapy outcomes are significantly correlated with lower risk scores. Therefore, we can conclude that immunotherapy is more likely to be beneficial for patients in the low-risk group. Together, the prognostic risk score model may be helpful in identifying patients' TIME and forecasting their response to immunotherapy.

### 3.7. Construction of ceRNA Networks

**Figure 8:**
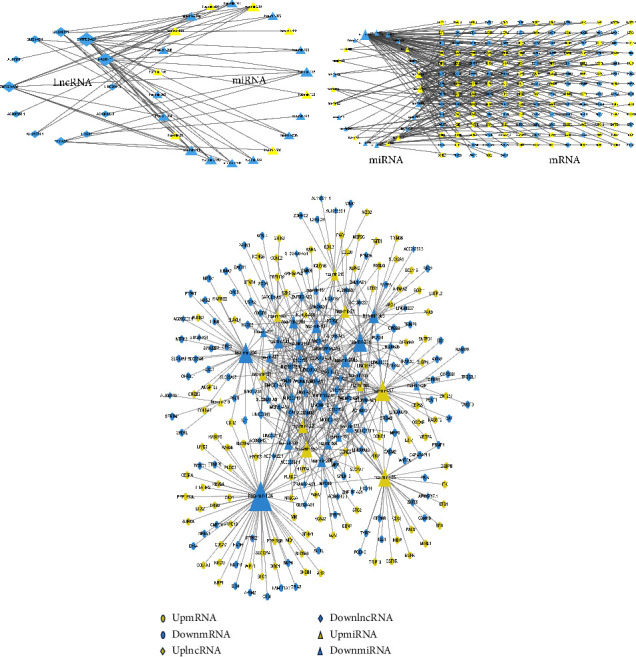
Construction of the ceRNA network. (a) Interaction network diagram between lncRNA and miRNA; (b) interaction network diagram between mRNA and miRNA; (c) ceRNA network diagram of lncRNA-miRNA-mRNA. Yellow means upregulation, blue means downregulation.

It is generally known that miRNA can influence mRNA expression via binding to mRNA. As a ceRNA, lncRNA can also control the expression of mRNA by competitively binding to miRNA, influencing the occurrence and progression of cancer. To learn more about the regulatory role of CR-lncRNA at the gene level, we firstly predicted the target miRNAs of the aforementioned prognostic CR-lncRNAs using the website miRcode, yielding 23 differential miRNAs (Figure 8(a)). The target miRNAs identified above were then used to predict the target mRNAs via the miRDB, miRTarBase, and TargetScan websites, and a total of 174 differentially overlapping mRNAs were discovered (Figure 8(b)). We created a lncRNA-miRNA-mRNA ceRNA network based on the results above (Figure 8(c)).

### 3.8. Functional Enrichment Annotation

**Figure 9:**
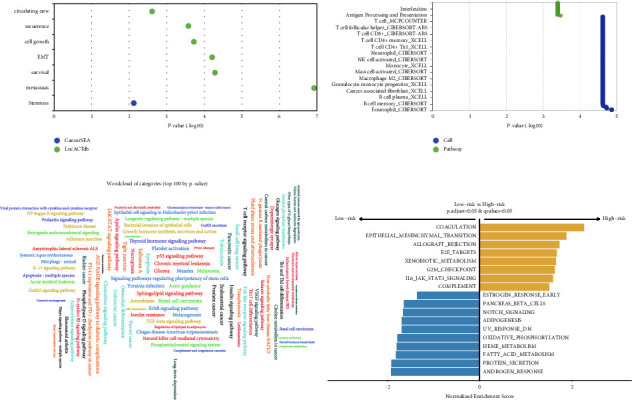
Functional enrichment analysis. (a-b) lncRNA enrichment analysis entry; (c) miRNA enrichment analysis entries; (d) GSEA enrichment analysis entries for high- and low-risk groups.

We discovered that the aforementioned prognostic CR-lncRNAs were considerably enriched in cell proliferation, metastasis, stemness, and EMT as well as being significantly related with a variety of immune cells by enrichment analysis (Figures 9(a) and 9(b)). The miRNA enrichment analysis revealed that the aforementioned miRNAs were significantly enriched in pathways involved in the development of cancer and immune inflammation, including the p53 signaling pathway, the JAK-STAT signaling pathway, the expression of PD-L1, the PD-1 checkpoint pathway in cancer, the chemokine signaling pathway, and other pathways (Figure 9(c)). The underlying biological pathways in patients in the high-risk and low-risk groups were then further investigated using GSEA analysis. According to the findings, the IL-6/JAK/ STAT3 signaling, E2f targets, and epithelial-mesenchymal transition (EMT) pathways were considerably enriched in the high-risk group. Patients in the low-risk group, on the contrary side, had significantly higher levels of pathways such oxidative ylation, protein metabolism, fat acid metabolism, and androgen response (Figure 9(d)).

### 3.9. Sensitivity Analysis of Antitumor Drugs

**Figure 10:**
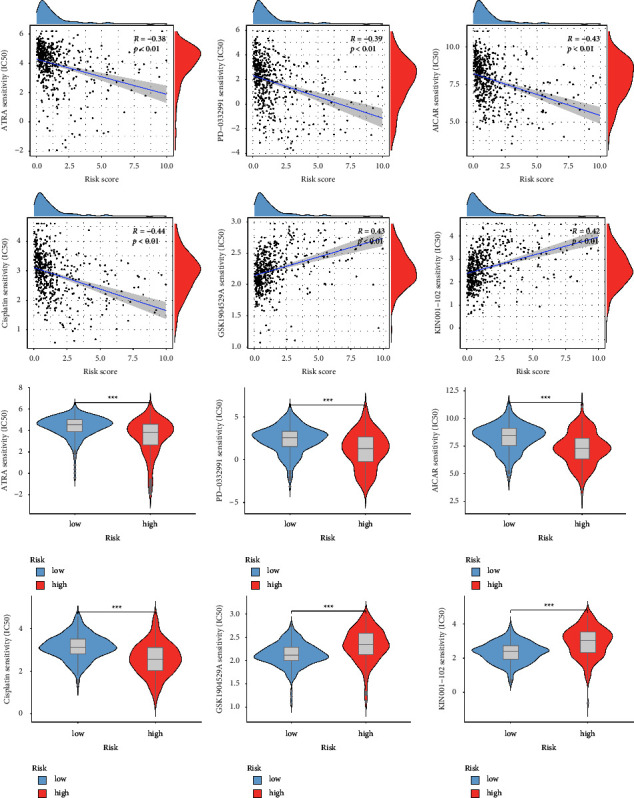
6 potential antitumor drugs-based on prognostic risk score model. ^*∗*^*P* value <0.05, ^*∗∗*^*P* value <0.01, ^*∗∗∗*^*P* value <0.001.

With the use of the “pRRophetic” R package, we acquired 6 potentially sensitive medications to help further direct the individualized treatment of ccRCC patients. Results showed that in the high-risk group, acadesine (AICAR), all-trans retinoic acid (ATRA), palbociclib (PD-0332991), and cisplatin were more sensitive, whereas in the low-risk group, GSK1904529A and KIN001102 were more sensitive (Figure 10).

## 4. Discussion

Using the TCGA and GEO datasets, this study investigated the expression differences of CRGS at the gene level between normal tissue and tumor samples, and further confirmed the expression variations of CRGS at the protein level in the HPA datasets. In ccRCC, the majority of CRGS were lowly expressed, and a survival study afterward indicated that most CRGS were protective genes in ccRCC patients. In addition, the genetic mutation data analysis confirmed that the genetic mutation of the above genes in ccRCC is relatively rare. The two subtypes of cuproptosis clusters were then established by consensus clustering-based on the expression of 19 CRGS, and further analysis proved that the subtype with high CRGS expression was substantially associated with higher survival. These findings imply that cuproptosis might be a therapeutic target for people with ccRCC. It is interesting to note that there were significant differences between the TIME of the two subtypes, with the subtype considerably downregulated in CRGS having a larger abundance of cytotoxic T lymphocytes (CTLs) infiltration but also more MDSC infiltration. It is well recognized that MDSC influence immunosuppressive tolerance through a variety of methods as significant elements of the milieu that suppresses the immune response to cancer. Numerous studies have proven that MDSC, in particular, suppress the T-cell immunological response by creating a lot of reactive oxygen species (ROS) [43–45]. Also, MDSCs have been linked to a number of tumor-related events, including angiogenesis, treatment resistance, and metastasis [46]. This may also explain why cluster B subtypes have lower survival rates and higher CTLs infiltration. Notably, subsequent GSVA analysis also supported the finding that patients with the cluster B subtype had substantial ROS PATHWAY enrichment. The outcomes of the TIDE online tool also revealed that patients with the cluster B subtype responded to immunotherapy less favorably. The statistics shown above clearly demonstrate that cuproptosis is highly related to the prognosis and immunotherapy of patients with ccRCC, opening up new research directions.

LncRNA, which acts as master regulator of gene expression, has been linked to a number of cancers and can be used independently to predict a patient's prognosis and make a diagnosis of the disease [40–42]. By using WGCNA, we were able to recognize CR-lncRNA. Subsequently, prognostic characteristic genes were further screened using univariate Cox regression analysis, log-rank test, LASSO regression, and RF. Finally, using multiple stepwise Cox regression, an optimal prognostic risk score model made up of 13 CR-lncRNAs was constructed. The model has strong predictive performance and consistency, as indicated by the ROC curve and the C index. Furthermore, it was discovered that the CR-lncRNA-based prognosis models developed using WGCNA and various machine learning algorithms were typically superior to some current models when compared to some lncRNA-based prognostic models developed in the TCGA-KIRC cohort in recent years.

Besides that, we investigated the relationship between predictive risk scores and clinicopathological characteristics and discovered that there was a substantial relationship between risk scores and clinicopathological variables in ccRCC. Furthermore, studies showed a significant positive correlation between the risk score and the tumor's aggressiveness, with the greater the risk score, the higher the tumor grade and stage. Subsequent analysis of immune checkpoint expression and immune infiltration analysis confirmed that, except for PD-L1 and PD-L2, the remaining immune checkpoints were more highly expressed in the high-risk group, and the infiltration abundance of MDSC was also higher. This demonstrates that patients with higher risk scores are more likely to produce an immunosuppressive microenvironment, enabling tumor cells to elude the immune system's surveillance and promoting the growth and development of malignancies. Furthermore, evidence that patients with greater risk scores had a worse response to immunotherapy came from the TIDE study. We conducted the GSEA analysis to investigate the mechanism underlying this difference. Pathways including EMT and IL6 Jak Stat3 Signaling were discovered to be considerably enriched in the high-risk group. Studies have already shown that activating the EMT pathway can promote tumor cell infiltration, tumor migration, and metastasis. It can also cause the formation of an immunosuppressive microenvironment, which helps tumor cells to escape the immune system [47, 48]. Meanwhile, the IL-6/JAK/ STAT3 Signaling pathway is overactivated in many forms of cancer, and it is implicated in driving cancer cell proliferation, invasion, and metastasis, as well as interacting with TIME to inhibit antitumor immune responses [49]. Moreover, it has been shown that the Stat3 transcription factor in the Stat3 signaling pathway can increase the expression of S100A8 and S100A9, preventing dendritic cell (DC) differentiation and stimulating the accumulation of MDSC, which in turn mediates the immunosuppressive effects [50]. According to the results above, there may be a connection between the activation of the aforementioned pathways and the differences in TIME and immunotherapy responses amongst different risk subgroups.

We developed a lncRNA-miRNA-mRNA ceRNA network to more thoroughly elucidate the regulatory role of CR-lncRNA at the gene level. Following enrichment analysis, it was discovered that the aforementioned lncRNAs and miRNAs were strongly linked to tumor development, metastasis, prognosis, cell proliferation, and TIME. AICAR, ATRA, PD-0332991, Cisplatin, GSK1904529A, and KIN001-102 were among the six possible anticancer medications that were tested using drug sensitivity analysis. And research has shown that ATRA can enhance the survival of tumor-specific CD8 T cells and upregulated MHC I expression in tumor cells to function as anti-tumor immunity [51–53]. Additionally, it can also promote MDSC differentiation and maturation, which in turn lowers their population, triggering the immune system to inhibit tumor growth [54]. A highly selective CDK4/6 inhibitor known as PD-0332991 has been shown to have antiproliferative effects in a variety of malignancies, including renal cell carcinoma and liver cancer [55, 56].

This study has some relative merits overall. First off, the CR-lncRNA-based prognostic model created by the WGCNA and several machine learning algorithms can successfully predict the prognosis of ccRCC patients. It offers greater prediction performance and consistency when compared to several other lncRNA-based models already in use. Significant relationships between the model, TIME, and immunotherapy were also discovered in the final research. There are, however, some restrictions-based on bioinformatics analysis, and multicenter prospective studies are still required for validation in the latter phase, which is also the main objective of our future research work.

## 5. Conclusion

We explore the potential function of CRGS in ccRCC after a thorough investigation. Based on CR-lncRNA, a model for prognostic risk scoring was developed. This model can distinguish TIME, predict the effectiveness of immunotherapy, and provide great and independent prognostic performance in ccRCC patients, allowing for more personalized treatment. For upcoming ccRCC research, it offers fresh perspectives and ideas.

## Data Availability

The datasets used to support the findings of this study are publicly available in the GEO database (https://www.ncbi.nlm.nih.gov/geo/) and TCGA database (https://portal.gdc.cancer.gov/).
